# Commentary: Mitochondrial encephalomyopathy with lactic acidosis and stroke-like episodes with an *MT-TL1 m.3243A*>*G* point mutation: neuroradiological features and their implications for underlying pathogenesis

**DOI:** 10.3389/fnins.2023.1173654

**Published:** 2023-04-17

**Authors:** Josef Finsterer, Sounira Mehri

**Affiliations:** ^1^Neurology and Neurophysiology Center, Vienna, Austria; ^2^Biochemistry Laboratory, LR12ES05 “Nutrition-Functional Foods and Vascular Health”, Faculty of Medicine, Monastir, Tunisia

**Keywords:** MELAS, mtDNA, heteroplasmy, respiratory chain, stroke-like episode

## Introduction

We read with interest the article by Zheng et al. on the imaging characteristics and their implications on the underlying pathophysiology in 59 imaging studies from 24 patients with mitochondrial encephalopathy, lactic acidosis, and stroke-like episode (MELAS) syndrome due to the most common mtDNA variant responsible for MELAS, m.3243A > G (Zheng et al., 2023). It was found that stroke-like lesions (SLLs), the morphological equivalent of stroke-like episodes (SLEs) were most commonly located in the occipital lobe (63%), followed by the parietal (54%) and the temporal (51%) lobes (Zheng et al., 2023). Two thirds of patients presented with basal ganglia calcification, cerebral or cerebellar atrophy, and with dilatation of cerebral arteries supplying SLLs (Zheng et al., 2023). It was concluded that imaging abnormalities in MELAS are disease stage-dependent and that polymorphic lesions in a single examination are suggestive of MELAS (Zheng et al., 2023). The study is excellent but has limitations that raise concerns that should be discussed.

It should be mentioned how many patients had epilepsy, how many had seizures prior to onset of SLEs, and how many had seizures during the presence of the SLL. Because seizures can trigger SLLs (Finsterer and Zarrouk, 2022), it is crucial to know in how many of the included patients the individual history was positive for epilepsy or seizures and how many had epileptiform discharges on electroencephalography (EEG). Knowing how many patients had seizures is crucial for interpretation of cerebral imaging abnormalities, as seizures can manifest with hyperintensities on diffusion-weighted imaging (DWI; Qi et al., 2022). In addition, it should be reported how many were regularly taking anti-seizure drugs (ASDs) and what type of ASDs these patients were taking.

It would be beneficial to apply additional MRI modalities. Because oxygen-extraction fraction (OEF) MRI can demonstrate reduced extraction of oxygen from erythrocytes within SLLs (Xie, 2014), it is crucial to apply this modality to MELAS patients. On OEF-MRI, SLLs appear hypointense. Extraction of oxygen from blood is reduced because it cannot be sufficiently utilized within mitochondria due to impaired respiratory chain function. Because SLLs typically show hypometabolism on fluor-deoxy glucose (FDG) positron emission tomography (PET; Kim et al., 2001), it is recommended to apply FDG-PET to MELAS patients with acute SLLs to document impairment of the mitochondrial oxidative metabolism within the area of a SLL.

Imaging findings should be related to heteroplasmy rates of the m.3243A > G variant. Knowing heteroplasmy rates from clinically affected and unaffected tissues is crucial not only for assessing the prognosis of an affected individual and for genetic counseling but also for correlating the degree of damage with the amount of mutated mtDNA.

Because the heart can be affected in MELAS patients (Finsterer and Zarrouk-Mahjoub, 2020), it is essential to consider that these patients can suffer ischemic stroke due to cardio-embolism from atrial fibrillation, heart failure, dilated cardiomyopathy, Takotsubo syndrome (TTS), or left ventricular hypertrabeculation. In addition, MELAS patients can carry classical cardio-vascular risk factors, such as arterial hypertension, hyperlipidemia, diabetes, or can be smokers.

Overall, the interesting study has limitations that call the results and their interpretation into question. Clarifying these weaknesses would strengthen the conclusions and could improve the study. Interpretation of cerebral lesions in MELAS should not only focus on SLLs but also on other imaging abnormalities, such as cardio-embolic ischemic stroke, bleeding, calcifications, atrophy, cysts, the toenail sign, and seizures equivalents.

## Author contributions

JF: design, literature search, discussion, first draft, critical comments, and final approval. SM: literature search, discussion, critical comments, and final approval. Both authors contributed to the article and approved the submitted version.

## References

[B1] Finsterer J. Zarrouk S. (2022). Ischemic and metabolic stroke can co-occur in m.3243A>G carriers: A case report. Cureus 14, e25705. 10.7759/cureus.25705 35812548 PMC9260700

[B2] Finsterer J. Zarrouk-Mahjoub S. (2020). The heart in m.3243A>G carriers. Herz 45, 356–361. 10.1007/s00059-018-4739-6 30128910

[B3] Kim H. S. Kim D. I. Lee B. I. Jeong E. K. Choi C. Lee J. D.. (2001). Diffusion-weighted image and MR spectroscopic analysis of a case of MELAS with repeated attacks. Yonsei Med. J. 42, 128–133. 10.3349/ymj.42,1.128 11293491

[B4] Qi Z. Li Z. Gao Q. Dong L. Lin J. Peng K.. (2022). Characterizing cerebral imaging and electroclinical features of five pseudohypoparathyroidism cases presenting with epileptic seizures. Behav. Neurol. 2022, 8710989. 10.1155/2022/8710989 35992960 PMC9391127

[B5] Xie S. (2014). MR OEF imaging in MELAS. Methods Enzymol. 547, 433–444. 10.1016/B978-0-12-801415-8.00021-7 25416369

[B6] Zheng H. Zhang X. Tian L. Liu B. He X. Wang L.. (2023). Mitochondrial encephalomyopathy with lactic acidosis and stroke-like episodes with an *MT-TL1 m.3243A*>*G* point mutation: Neuroradiological features and their implications for underlying pathogenesis. Front. Neurosci. 16, 1028762. 10.3389/fnins.2022.1028762 36685235 PMC9853426

